# Designing a theory-informed, contextually appropriate intervention strategy to improve delivery of paediatric services in Kenyan hospitals

**DOI:** 10.1186/1748-5908-8-39

**Published:** 2013-03-28

**Authors:** Mike English

**Affiliations:** 1 KEMRI-Wellcome Trust Research Programme, P.O. Box 43640, Nairobi 00100, Kenya; 2 Nuffield Department of Medicine and Department of Paediatrics, University of Oxford, Oxford, UK

## Abstract

**Background:**

District hospital services in Kenya and many low-income countries should deliver proven, effective interventions that could substantially reduce child and newborn mortality. However such services are often of poor quality. Researchers have therefore been challenged to identify intervention strategies that go beyond addressing knowledge, skill, or resource inadequacies to support health systems to deliver better services at scale. An effort to develop a system-oriented intervention tailored to local needs and context and drawing on theory is described.

**Methods:**

An intervention was designed to improve district hospital services for children based on four main strategies: a reflective process to distill root causes for the observed problems with service delivery; developing a set of possible intervention approaches to address these problems; a search of literature for theory that provided the most appropriate basis for intervention design; and repeatedly moving backwards and forwards between identified causes, proposed interventions, identified theory, and knowledge of the existing context to develop an overarching intervention that seemed feasible and likely to be acceptable and potentially sustainable.

**Results and discussion:**

In addition to human and resource constraints key problems included failures of relevant professionals to take responsibility for or ownership of the challenge of pediatric service delivery; inadequately prepared, poorly supported leaders of service units (mid-level managers) who are often professionally and geographically isolated and an almost complete lack of useful information for routinely monitoring or understanding service delivery practice or outcomes. A system-oriented intervention recognizing the pivotal role of leaders of service units but addressing the outer and inner setting of hospitals was designed to help shape and support an appropriate role for these professionals. It aims to foster a sense of ownership while providing the necessary understanding, knowledge, and skills for mid-level managers to work effectively with senior managers and frontline staff to improve services. The intervention will include development of an information system, feedback mechanisms, and discussion fora that promote positive change. The vehicle for such an intervention is a collaborative network partnering government and national professional associations. This case is presented to promote discussion on approaches to developing context appropriate interventions particularly in international health.

## Background

Dramatic improvements in child, newborn, and maternal survival by 2015 are major global development goals (MDGs 4 & 5, [1]). Unfortunately many African countries are not on track to achieve them [2] with a major obstacle being inadequate delivery of evidence-based, essential interventions that are one aspect of high-quality care [3,4]. Improving their delivery requires broad strengthening of national health systems [5-7] as a multitude of factors may influence how services are actually delivered including: financing, human resources, governance and information systems in addition to more traditional concerns over knowledge, skills, and availability of technologies [8,9].

The setting focused on here is the district hospital. These are typically linked to a network of primary care and community-based health services [10]. In Africa, they consume approximately 50% of financial and human resources and are, as the training ground for most health workers, a major influence on the value of the human capital within health systems [11-13]. Here, both basic and slightly more complex cost-effective interventions are provided in a multi-professional setting that concentrates skills and resources [3,10,14,15]. Unfortunately, however, district hospitals currently perform poorly in delivering high quality, essential services [16-24]. For example, ill newborns or children with severe malnutrition are often not fed appropriately and drug dose errors remain common [25].

To improve this situation past intervention efforts in low-income settings have often been guided by simple, mechanistic views of contexts and systems [26]. For example, simply increasing the supply of new guidelines or training is common, even though findings from higher income settings indicate that alone these usually fail to change performance [26]. Increasingly therefore, broader, more system-oriented interventions addressing the many important influences on provider or user behavior [27-29] are felt to be needed [8]. This research must be conducted in, and designed for, real life settings.

This paper describes an approach to developing a system-oriented intervention to improve services for children in Kenyan district hospitals. It is an effort to open up, to a degree, the black box between proposed treatment and desired effect [30]. Before presenting the approach and proposed intervention, however, key aspects of the current Kenyan context are summarized.

### Key aspects of the Kenyan context

Kenya remains a low-income country with a 2010 per capita income of $810 and 46% of the population living below the poverty line. Hospital services are largely provided through the public sector in rural areas and there are relatively few health providers for Kenya’s population of 40 million (1 medical doctor and 12 nurses/10,000 population respectively). Although there have been improvements in child survival it is estimated that 74/1000 children die before their fifth birthday and maternal mortality stands at 390/100,000 live births. Kenya’s more recent health policy context has much in common with that in many Anglophone African countries. The late 1980’s saw the adoption of New Public Management inspired policies aimed at introduction of performance management and advocacy for the ‘empowerment’ of managers [31]. More recent policy initiatives, including those espoused in Kenya’s new constitution [32], suggest a continued devolution of powers. This is, however, within a country in which the total public per capita spending on health is approximately $20 per annum.

### Kenyan district hospitals—basic organizational context

District hospitals vary in size from those with 50 to 80 inpatient beds to those with 300 to 500 beds. Administrative responsibility lies with a hospital management team comprising persons in charge of administration, nursing, pharmacy, and allied health services. This team is led by a medical superintendent supported by only one or two professional administrators for the entire hospital. In recent surveys, these senior healthcare staff self-reported that they felt poorly prepared for their administrative roles [33]. Traditionally, neither basic nor post-graduate clinical or nursing courses have addressed management training to any significant degree (although this is beginning to change).

Within hospitals, those in charge of different clinical service units or departments occupy the middle level of management. The lead clinician may have a higher qualification in a medical specialty (*e.g.*, paediatrics) but in smaller hospitals a general medical practitioner with as little as one year of work experience may take charge of a department [34]. While nurses leading departments tend to have more years work experience, few at this level have higher training in a clinical specialty. Job descriptions, if available at all, are rarely explicit about service delivery or management responsibilities (unpublished observation). Relevant professional associations have no significant history of providing professional training aimed at service delivery improvement. Despite this, the clinicians and nurses leading service units have significant management roles. For example, they are expected to plan and advocate for departmental resources though they are unlikely to have direct control over a specific budget. Such individuals are also the primary leaders and supervisors of their team of frontline health workers with whom they deliver routine services.

The nature of frontline clinicians varies with hospital size and location. Larger hospitals with a post-graduate trained medical specialist as lead clinician typically have doctors doing their one-year internship after basic training, clinical officers (diploma level clinicians), clinical officer interns, or a mix of all these groups [34]. In the largest district hospitals, one or perhaps two post-internship general medical officers may also be part of the team. In smaller hospitals, a lead general medical officer is likely to be supported by clinical officers and clinical officer interns alone. Frontline nurses predominantly have a certificate or diploma in general nursing, degree level nurses remain very few in frontline positions [35].

### Prior service improvement efforts

Poor access to knowledge products is not now the major underlying problem in Kenya. In earlier work (in 2002 and 2006), it was clear that guidelines were rarely found in district hospitals, and no major efforts had actively been made to implement their recommendations [17,34]. In 2006, however, national paediatric and neonatal guidelines were developed [36] and 10,000 copies were distributed by the end of 2007 by the Ministry of Health. These initial guidelines were developed as part of a trial of two alternative implementation strategies. This trial demonstrated that combining provision of guidelines with skill-based training, external support supervision, local facilitation, and feedback every six months on progress enhanced uptake of recommended practices compared to provision of guidelines linked to short, didactic training, and written feedback every six months [37]. Positive results of implementation studies and demand for guidelines from practitioners resulted in their revision in 2010 [38] with a further 12,000 copies disseminated by mid-2012. In addition, the skill-based course (ETAT+) used to promote guideline uptake [36] proved very popular. This course became progressively more widely utilized in Kenya from 2008 also in response to demand, with over 2,500 health workers given in-service training by 2013 (still less than 10% of the public workforce). The course was also adopted as part of pre-service and post-graduate paediatric training in the country’s largest medical school from 2008 [39].

However, work also indicated significant variation in uptake of best practices even within hospitals receiving the more comprehensive intervention approach. In work attempting to explain these observations the level of engagement of senior and particularly mid-level clinical managers was important [40]. So too was the relationship between implementers/supervisors and hospitals and the strength of the ‘soft-contract’ between these two parties. Although the Ministry of Health has also attempted to promote supervision of district hospitals in recent years, the approach still tends to focus on procedural adherence [41]. Yet, efforts to improve paediatric service delivery must move to scale and be adaptable to changing conditions and potentially other clinical disciplines. There is thus potential for a third, locally credible party to take on roles to work with professionals to improve services. In many higher income settings such a third party role has been adopted, at least in part, by professional associations.

## Methods

The overarching approach to developing the intervention was based on four main strategies: an iterative and reflective process aiming to draw lessons from the author’s prior research [10,17,34,37,39,40,42-44] in Kenya, accompanying observations of the Kenyan health system over a period of many years, and multiple informal discussions with other researchers, policy makers, senior professionals and district hospital practitioners with the aim to distill root causes for the problems with service delivery observed; developing a broad set of intervention options to address these problems identified from literature, discussions, and experience; a search of literature for theory that provided the most appropriate basis for intervention (and evaluation) design; and repeatedly moving backwards and forwards between identified causes, proposed interventions, identified theory, and knowledge of the existing context to develop an overarching intervention that seemed the ‘best fit’ and was deemed feasible, likely to be acceptable, and potentially sustainable. Throughout this process, the following questions guided thinking: Why is widely available practice guidance not reliably being used in district hospitals? What aspects of the current health system configuration are supporting or failing to support improved service delivery? On whom does service delivery improvement most depend? How does the system supporting service delivery improvement need to change, and at what system levels, to promote improvement in the absence of significant changes in material or human resources? Which interventions would fit best into existing structures and foster acceptable, feasible, and potentially sustainable evolution of the health system supportive of improved care? Potential intervention strategies were further refined through ongoing discussions with policy makers, senior professionals, and colleagues. This process was conducted during and in anticipation of a continued slow shift away from a highly centralized public health system.

## Results and discussion

As indicated above, the approach was iterative moving between problems, potential solutions, and theory while considering context all the time. In presenting the outcome of this process, I first outline the root causes for poor service delivery, link these to general needs, and then describe the specific aspects of the intervention strategy proposed. This is followed by an explanation of how recent broad theories on implementation informed these decisions linked to the identification of actors central to the overall strategy. Finally, key underlying themes guiding the selection of intervention components or the process of implementation are discussed.

### Root causes of poor service delivery

Practices have improved with the guideline dissemination efforts outlined above and other initiatives. However, care is still often inconsistent with evidence-informed, ‘best-practice’ recommendations [45,46]. Central problems identified in moving beyond dissemination to widespread implementation are that:

1. Those expected to have the responsibility for overseeing and promoting the provision of best practice care in paediatric service units have not yet taken ownership of this role.

2. Those expected to perform this role are often poorly prepared or equipped for it, while they work in a system that provides little support for such roles and in which they are often both professionally and geographically isolated.

3. The health system currently generates very little useful information for routinely monitoring or understanding service delivery practice or outcomes.

Consequently an intervention to address these core challenges should:

1. Help shape an appropriate role for key paediatric service delivery personnel and promote their identification with and ownership of this role.

2. Provide health workers assuming such roles with the necessary understanding, knowledge, skills, and ongoing support to:

a. Work with senior managers to negotiate for and mobilize necessary resources and improve organization of services where possible and,

b. Work with frontline staff to improve their knowledge, skills, and practices where required.

3. Support the development of an information system, feedback mechanisms, and discussion fora that promote the appropriate use of information for service improvement at all levels of the health system

An effective intervention approach should address these challenges and at the same time be feasible, acceptable, potentially sustainable beyond any research effort, and designed for the existing context and its readiness for change.

### Specific intervention activities

The major structural and organizational approach will be the development of a hospital network linked by a central hub. This network structure will aim ‘to improve clinical care and service delivery using a collegial approach to identify and implement strategies’ (adapted from Haines 2012 *et al.*[47]). It will work as a partner to government and align itself formally with the Kenya Paediatric Association. This association is beginning to assume some responsibility for promoting service standards and supporting continuous professional development for health workers providing care to children. The network will:

1. Develop consensus-based agreements on service delivery priorities and goals for hospitals that are feasible and appropriate to the level of resources available. Such goals will be used to develop indicators that will form the basis of assessment and feedback. These can be revised over time. At present in Kenya such service delivery indicators are largely absent or too crudely defined to be useful or measurable.

2. Work with government to foster the development of improved hospital information systems that will allow collection of data on key indicators of service delivery over time. At present in Kenya health information systems are unable to provide reliable data on hospital services or outcomes [46].

3. Work with government to collate and analyze relevant service delivery indicator data and use these data to provide feedback to hospitals, their staff, and government on a regular basis. The network will emphasize the importance of feedback as a tool to support improvement rather than identify those who should be sanctioned. (This work will enhance the currently very limited capacity to analyze service delivery information in government in Kenya).

4. Provide training for mid-level clinician/nurse managers to enhance their knowledge and understanding of their roles and responsibilities when leading service provision. Training will also be provided in:

a. Basic elements of clinical leadership with a focus on how to promote collective action within their service delivery teams.

b. Simple quality improvement techniques, use of clinical audit as a tool for reflecting on service organization and practice, and basic problem solving techniques such as the use of process mapping.

5. Provide support and mentorship for mid-level clinician/nurse managers. This will include efforts to enhance technical clinical/nursing knowledge where necessary as well as providing mid-level clinician/nurse managers with a forum for discussing challenges they face in their managerial roles.

6. Promote identification of successful strategies for improving services (exemplars) and their dissemination and continued ‘testing’ within the network as part of shared and ‘evolutionary’ learning.

7. Recognize and make visible to hospitals within the network and to policy makers any significant achievements.

**Figure 1:**
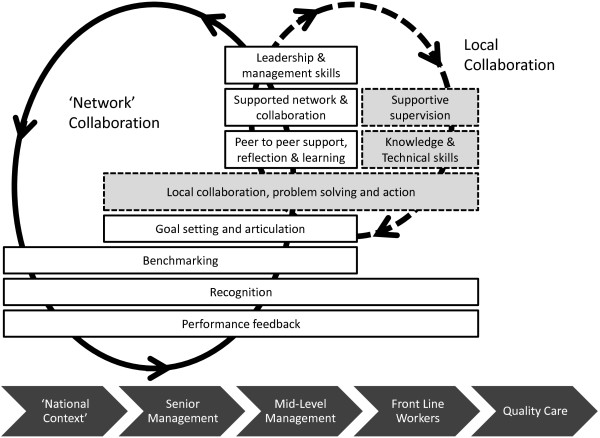
**Schematic representing the direct aspects of intervention.** Unshaded boxes represent intervention aspects that are the focus of the network and higher-level feedback and learning. Indirect aspects of the intervention that might result from lower, hospital level collaboration catalyzed by the direct intervention are presented in shaded boxes. Intervention components may affect one or more organizational levels while ultimately intended to improve quality of care. The dynamic and iterative nature of the intervention comprised by the network collaboration is suggested by the complete circular line, while the iterative nature of local, internal effects that may result is suggested by the broken line. Such dynamic effects may be synergistic, as is hoped, or antagonistic.

To deliver these intervention components the network will be supported by a clinical coordinator, an administrator and a small ‘information team’ for at least three years. The aim will be to provide feedback on performance against agreed indicators to government, hospitals and their staff approximately every two months. Network meetings for paediatric mid-level managers from all hospitals will be supported. At present, there are no such approaches to performance feedback in Kenya and no fora focused on reviewing or discussing service improvement. In the first year, three network meetings are planned, with two in the second year followed then by annual meetings. The network coordinator will conduct visits twice a year to hospitals accompanied by government officers and/or peers from within the network. These visits will include on-site performance review and problem solving meetings and help develop approaches to providing support supervision within the government system. The coordinator will also be available to provide telephone or email advice as requested. Complementing network meetings and visits the potential for developing a low-cost, confidential, internet community of practice will be explored to allow network hospitals to interact continuously. Here, participants will be able to discuss performance data, share ideas on improvement strategies and experiences, and seek the advice of peers, the coordinator, and potentially other mentor figures. The targets of these planned activities and their hoped for synergistic or catalytic effects are illustrated in Figure 1.

### How prior research and theory informed intervention design

There is an increasing body of research that has investigated specific interventions targeting change in health worker behaviors using, for example, financial incentives, computer aided decision support, or mobile phone text messaging reminders [48-51]. Other interventions examined are intended to influence health worker groups, for example, through training, team-based audit and feedback, or expert outreach [52-54]. Much of this work has now been synthesized in the form of systematic reviews and some of these are introduced in Table 1. A growing number of examples of multi-faceted interventions that aim to change practices within organizationally-defined service delivery units or facilities as a whole are also reported [37,55-57]. The intervention planned will therefore employ-specific, non-financial approaches that lend themselves to combination within a network approach and that are supported by existing evidence: educational meetings; educational outreach visits; local opinion leaders, printed educational materials (already in circulation); and audit and feedback. While information and communication technologies may be used to assist joint learning (see below), hospitals in Kenya do not have inpatient electronic medical records, and computerized decision support is not yet feasible.

**Table 1:** Summary of conclusions from systematic reviews of experimental studies examining specific intervention approaches to change practitioner behavior or patient outcomes

**Managerial supervision (LMIC) [**[58]**]**	**Little evidence for positive effects in primary healthcare settings, but poorly studied**
Clinical pathways [59]	Clinical pathways are associated with reduced in-hospital complications and improved documentation without negatively impacting on length of stay and hospital costs.
Organizational infrastructures [60]	Only one low-quality study.
Strategies to change organizational culture [48]	No studies that fulfilled the methodological criteria for this review.
External inspection of compliance with standards	Only two studies identified for inclusion in this review, no firm conclusions could therefore be drawn about the effectiveness of external inspection on compliance with standards.
Continuing education meetings and workshops [52]	Educational meetings, alone or combined with other interventions, can improve professional practice and healthcare outcomes for the patients. The effect is most likely to be small and similar to other types of continuing medical education, such as audit and feedback, and educational outreach visits. Strategies to increase attendance at educational meetings, using mixed interactive and didactic formats, and focusing on outcomes that are likely to be perceived as serious may increase the effectiveness of educational meetings. Educational meetings alone are not likely to be effective for changing complex behaviors.
Educational outreach visits (EOV) [54]	EOVs alone or when combined with other interventions have effects on prescribing that are relatively consistent and small, but potentially important. Their effects on other types of professional performance vary from small to modest improvements, and it is not possible from this review to explain that variation.
Local opinion leaders [61]	Opinion leaders alone or in combination with other interventions may successfully promote evidence-based practice, but effectiveness varies both within and between studies. These results are based on heterogeneous studies differing in terms of type of intervention, setting, and outcomes measured. In most of the studies the role of the opinion leader was not clearly described, and it is therefore not possible to say what the best way is to optimize the effectiveness of opinion leaders.
Printed educational materials (PEMs) [62]	The results of this review suggest that when compared to no intervention, PEMs when used alone may have a beneficial effect on process outcomes but not on patient outcomes. Despite this wide of range of effects reported for PEMs, clinical significance of the observed effect sizes is not known. There is insufficient information about how to optimize educational materials. The effectiveness of educational materials compared to other interventions is uncertain.
Public release of performance data [63]	The small body of evidence available provides no consistent evidence that the public release of performance data changes consumer behavior or improves care. Evidence that the public release of performance data may have an impact on the behavior of healthcare professionals or organizations is lacking.
Paying for performance (LMIC) [64]	The current evidence base is too weak to draw general conclusions; more robust and also comprehensive studies are needed.
Audit and feedback [53]	Audit and feedback generally leads to small but potentially important improvements in professional practice. The effectiveness of audit and feedback seems to depend on baseline performance and how the feedback is provided. Future studies of audit and feedback should directly compare different ways of providing feedback.
Inter-professional education (IPE) [65]	This updated review found six studies that met the inclusion criteria, in contrast to our first review that found no eligible studies. Although these studies reported some positive outcomes, due to the small number of studies, the heterogeneity of interventions, and the methodological limitations, it is not possible to draw generalizable inferences about the key elements of IPE and its effectiveness.

However, a focus on activities risks neglecting wider thinking on how to effect change for which a variety of models now exist. These include those framed around individual responses linked to guideline adoption such as that of Rogers [66]. This well-known model suggests that sub-populations of individuals exist who are more (innovators and early adopters) or less (laggards) ready to change at a personal level. One implication is that to get wholesale change efforts need to be sustained. Other models take a more organizational perspective [67], integrate individual and organizational ideas [68], or remind us of the hierarchical nature of health systems and that change, or lack of it, may be the consequence of influences at multiple levels [28,69]. Common to most models is recognition that change is not uniform. Rather, it is a complex social process and that active pursuit of change is required [70-72]. While it is beyond the scope of this article to examine this broad field these theories remind us that:

‘A key principle of systems thinking and systems change [is that] cause and effect are not necessarily close in time or space and may be affected by multiple other systems or subsystems’ [69].

‘The interrelationship of system components requires one to design parallel and reinforcing elements within the system to effect meaningful change’ [69].

The intervention strategy therefore also draws on work suggesting how to produce wider, system effects. Recently, theoretical frameworks have been developed that draw many other theories or empiric findings together. Not surprisingly, these deliver a number of overlapping findings. In this work they were used to explore how and why potential intervention activities might be valuable in influencing hospital practice change. This helped identify those felt to address core problems and that might both fit the context and support the overall effectiveness of a package of activities.

The Consolidated Framework for Implementation Research (CFIR) was developed to ‘offer an overarching typology to promote implementation theory development and verification about what works’ [73]. It was not specifically aimed at informing the design of interventions but identifies five major domains, each composed of multiple more specific constructs (to total 39 in all), that theory suggests are likely to influence implementation success: intervention characteristics, outer setting, inner setting, characteristics of the individuals involved, and the process of implementation. In this framework, an important aspect of the intervention is how recommendations are themselves perceived and packaged. The process for developing these revised guidelines made clear use of evidence, engaged multiple stakeholders in the decision-making process and was undertaken with the authority of both government and credible local partners [38,74]. The guideline packaging is in the form of short, pocket-sized booklets containing algorithms and drug dose tables distributed to clinicians and nurses at no charge [39]. Although this presentation format has not been formally evaluated, there is evidence that clinicians carry them [45], and similar booklets have been adopted in two neighboring countries suggesting their suitability (unpublished data). Because the guidelines focus on cheap, basic interventions, the practices they suggest incur no financial cost to institutions [42]. The existing paediatric guidelines were developed, packaged, and introduced in ways that by these criteria would be expected to facilitate their adoption. Planned attributes of the intervention design linked to further specific constructs in the CFIR are: peer pressure, cosmopolitanism, promotion of networks and communications, use of goals and feedback, development of a learning climate, leadership engagement, enhancement of self-efficacy and others [73].

The Theoretical Domains Framework (TDF) [75] was developed ‘to simplify and integrate a plethora of behavior change theories and make theory more accessible to, and usable by, other disciplines’ [76]. The TDF was recently revised to comprise 14 domains of theoretical constructs relevant to health workers’ change in behavior: knowledge, skills, social/professional role and identity, beliefs about capabilities, optimism, beliefs about consequences, reinforcement, intentions, goals, memory attention and decision processes, environmental context and resources, social influences, emotions, and behavioural regulation [76]. Most pertinent to this intervention are a focus on skills, social/professional role identity, reinforcement, goals, and social influences, particularly on mid-level and senior managers.

**Figure 2:**
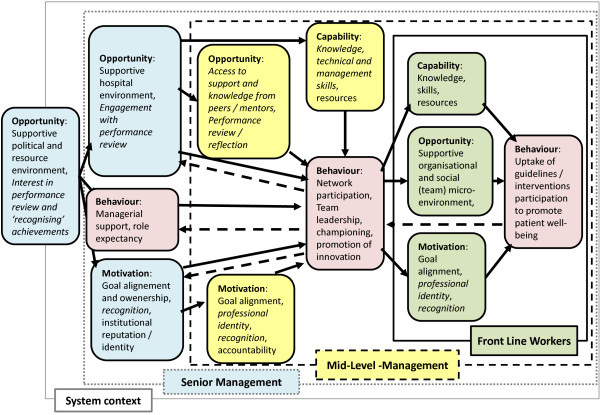
**Schematic representing key factors that are likely to influence the behavior of paediatric service leaders.** The key factors influencing behavior of service leaders is illustrate together with how this behavior and other factors at multiple levels impact on the behavior of frontline workers. This schematic adapts a framework proposed by Michie *et al.* that considers behavior to be influenced by capability, opportunity, and motivation [77] and extends this to consider the hierarchy of behavioral effects desired (pink shaded boxes) and the key intervention effects/levers anticipated to promote change at senior (blue shaded boxes) and mid-levels (yellow shaded boxes) of hospital management and among frontline (green shaded boxes) workers.

Most recently, Michie *et al.* have synthesized work on how theory might be used to promote implementation of existing guidance [77]. At the center of their thinking, Michie *et al.* place a ‘behaviour system’ involving three essential conditions: capability, opportunity, and motivation (that they term the ‘COM-B system’). This forms the hub of their Behaviour Change Wheel (BCW) around which are nine broad levers that may be utilized to effect change: education, training, persuasion, environmental restructuring, modeling, enablement, incentivization, coercion, and restriction. The effectiveness of these levers might, they suggest, be influenced by seven aspects of an enabling policy context: communication/marketing, guidelines, regulation, service provision, fiscal legislation, and environmental/social planning. The intervention developed has a core technical content supported at the highest policy level and the nature of approaches to their ‘communication and marketing’ has been described earlier. Behavioral levers felt to be feasibly and acceptably employed at scale include: relevant education and training, persuasion, modeling, enablement, and recognition as a key non-financial incentive. (See Additional file 1 for full illustration of links the BCW and the proposed intervention). An important and deliberate focus of intervention is individual service unit leaders, who are felt to have a pivotal role in delivering the ultimate outcomes. For this reason, a more specific articulation of their desired roles was undertaken (see below). However, it is also clear that in such a system-oriented approach the aim is to influence the behavior of multiple actors, synergistically, at different system levels (illustrated in Figure 2).

### Mid-level managers—pivotal agents of implementation

**Figure 3:**
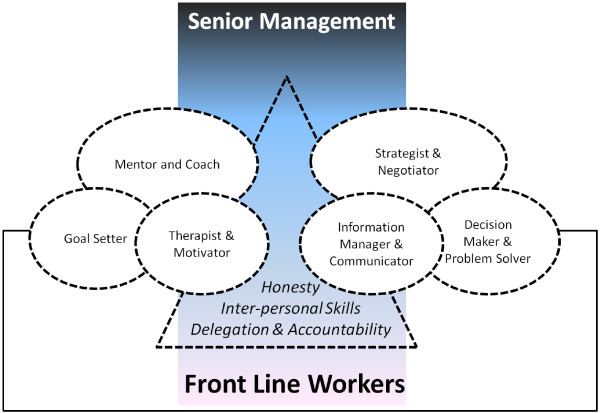
**Key roles and characteristics of effective mid-level managers in hospitals.** Key roles and characteristics of mid-level managers are presented encompassed in shapes with broken lines as the interface between senior management, represented by the vertical rectangle (who act largely through mid-level managers with relatively little interface with frontline workers) and the frontline workers, represented by the horizontal rectangle.

Above we indicated that departments are led and supervised by clinicians and nurses in the mid-level of management. In our previous work, we identified staff in such positions as key agents in the uptake and implementation of recommended practices [40], a finding consistent with wider work [78-80]. To understand the demands of these positions more fully, we recently reviewed literature on mid-level managers in hospitals to help us understand their expected, non-technical roles in an environment such as that in Kenya (for full report see Nzinga *et al*., in preparation). These roles can be grouped in nine overarching themes, six related to behavior as expected action and three areas of inter-personal behavior that support their management role (Figure 3). The former includes roles as: mentor and coach, linked to roles as goal setter and motivator and therapist, and strategist and negotiator, linked to roles as information manager and decision maker and problem solver. The latter includes demonstrating behavior that promotes good interpersonal relations, delegation and accountability, and honesty. These findings are broadly consistent with those of Birken *et al.*[81] who focused on mid-level managers’ roles implementing policy and practice from a more organizational perspective, condensing these roles into those of diffusion, synthesis, mediation, and marketing*.*

### Major themes underlying the proposed intervention approach

A critical consideration throughout the process of intervention design was developing a strategy that would enhance or complement existing or emerging features of Kenya’s health system. Such themes are now discussed.

### Developing shared goals and assessing progress

Any intention to improve service delivery is typically aimed at achieving some set of goals. This focus on goals resonates with the widespread adoption of ideas and practices from management sciences within health systems [82]. Much recent effort has gone into establishment of health systems targets and indicators and, for example, use of performance-based contracts for health managers linked to such indicators. Performance assessment in some form or another is now therefore widespread in Kenya. Often, however, the goals are simply imposed by the highest levels of government. The linked performance assessment approaches are then often poorly presented and managed and subsequently rejected. A focus on developing shared goals can, however, be a powerful way of forging an identity and common purpose. Yet there remain major questions over how to make the feedback of goal-linked performance data useful to individuals and at scale [83,84]. Nonetheless the ability to measure progress is likely key to engaging policy makers and hospitals’ managers (the outer and inner settings) in implementation efforts. Carefully utilized performance feedback may also be important for persuasion (*e.g.*, through recognition and bench-marking), enablement (*e.g.*, through demonstrations of what is achievable), and as an incentive (*e.g.*, through goal achievement and recognition).

### Building and utilizing skills and strategies associated with quality improvement

With origins in industrial process control, this field has evolved in healthcare as a specific attempt to embed and ground the healthcare improvement process in local contexts [85]. Reasonably generic techniques can be taught that benefit from local knowledge and innovation to help achieve overarching goals. In high-income settings, quality improvement is now accepted as an intrinsic part of most systems rather than a strategy to be tested for effectiveness. One programmatic approach is to develop ‘collaboratives’ to tackle key system challenges rapidly [86] (although evidence of their effectiveness is debated [87]). In Kenya, as in many low-income settings, service quality is a key policy objective, and quality improvement is part of the policy and management discourse though it is less often apparent in routine practice. However, improvement collaboratives can provide mentorship and support to organizations and key personnel and have been initiated in a number of low-income countries with positive results reported [88]. These approaches can provide relevant education and training for leaders of service units and thus mechanisms for them to persuade and enable frontline workers within hospital settings. Engaging a wider community of hospitals in linked efforts may strengthen the sense of a shared identity, may expose managers and individuals to the persuasive force of their peers, and may promote further relevant learning (see below).

### A focus on shared learning

Thinking on how to improve service delivery has benefited from recognition that health workers operate within informal, socially constructed contexts as well as formal organizational structures and physical contexts [71,89]. Approaches therefore increasingly recognize and take advantage of the considerable tacit knowledge held by health workers, formally bringing this to bear on efforts to improve services as part of organizational learning [90,91]. One such approach to harnessing the power of more socially-mediated shared learning may be through encouraging the development of communities of practice [92,93]. Notions of what these might comprise have evolved with a shift in thinking towards commissioned or managed forms rather than the self-generating forms that were initially explored [94]. These communities are linked by, or form around, a common challenge or goal of interest to members and operate in an open environment in which ideas and solutions that address the goal or challenge can be shared [94]. Communities can be relatively small or reach across departments, organizations, and countries. Used intentionally, they may foster innovative strategies to solve implementation or service delivery problems and provide a mechanism for sharing lessons learned within and across organizations. Because access to internet and smartphone technologies now penetrate even remote areas of Kenya, effectively established communities of practice could be a powerful social influence on both hospitals’ outer and inner settings and would provide relevant learning while modeling effective change efforts.

### Promoting collaboration

Although referring to community-focused activities, the words of Lasker and Weiss seem highly pertinent to organizations such as hospitals, which are one form of community. They state that ‘many of the problems that affect the health and well-being of people in communities [or hospitals] cannot be solved by any person, organization, or sector working alone…. Only by combining the knowledge, skills, and resources of a broad array of people and organizations can communities [or hospitals] understand the underlying nature of these problems and develop effective and locally feasible solutions to address them’ [95]. To achieve this it is argued that we should: empower individuals by getting them directly and actively involved in addressing problems that affect them; create bridging social ties that bring people together across dividing lines and build trust and a sense of community, and enable people to provide each other with various kinds of support; and create synergy—the breakthroughs in thinking and action that are produced when a collaborative process successfully combines the knowledge, skills, and resources of a group of diverse participants [95]. If we can achieve this, then health workers might: define approaches that they decide they need to work together on to accomplish something they cannot achieve alone; change the rules that inhibit progress and work toward changing these barriers; and change the system, where activities are expanded to work on systems change (Brindis and Wunsch, cited in [69]). Promoting collaboration is a key element of the process of the proposed intervention; between central implementers and hospitals (an aspect of the outer setting) and between leaders of service units and their senior managers and frontline workers (aspects of the inner setting). In Kenya, much may be gained by breaking down traditional hierarchies and promoting effective communication that are key barriers to service improvement [96]. New collaborative relationships will also be important in building trust and reciprocity and thus enhancing aspects of persuasion and modeling while providing an important basis for nurturing emergence of socially mediated, non-financial incentives.

### Summary

Some root causes of poor service delivery in Kenyan district hospitals have been identified and a broad, system-oriented strategy for intervention to improve services outlined. This strategy recognizes the need to provide a supportive outer and inner setting if frontline health workers are to take up best practice recommendations while positing a major role for mid-level managers as practice leaders and innovators within their own setting. While intervention-effect pathways are often presented in a simple, linear form, it is understood that in reality the intervention and the system it targets are complex [97]. Indeed, as the intervention itself will be delivered over time, it is expected that the ‘evolutionary pressures’ exerted by implementers and contexts will define its actual form [98]. This is perhaps especially likely because the intervention includes introduction of feedback loops that often produce non-linear and unanticipated effects, both positive and negative [99]. Planned evaluation (not described here) will therefore, characterize the context and process of intervention and their evolution to allow the ‘fidelity’ to original intervention aims to be determined. Nonetheless it is expected this intervention will (after Dixon-Woods *et al.*[100]): generate isomorphic pressures for hospitals to conform to improve practice; create a networked community with strong horizontal links that exerts normative pressures on members; reframes poor quality care as a social problem to be addressed through a professional movement combining ‘grassroots’ features with a vertically integrating program structure; employ several interventions that function in different ways to shape a culture of commitment to doing better in practice; harness data as a disciplinary force; and exert coercive forces (‘hard edges’) to promote improvement.

## Competing interests

The author declares that there are no conflicts of interests.

## Authors’ information

Mike English has worked in a research capacity for 17 years in partnership with the Kenya Medical Research Institute (KEMRI). Initially, he worked in a district hospital focusing on clinical and epidemiological research on paediatric and neonatal disease (notably malaria, pneumonia, meningitis and diseases of the newborn). In 2002, he undertook his first major piece of work with partners in the Ministry of Health examining routine paediatric and neonatal service delivery in hospitals across Kenya. As a result of the finding of poor quality care, he moved to Nairobi in 2004 with the aim of developing work to translate research findings into policy and practice. He engaged with the Ministry of Health, KEMRI and colleagues in the University of Nairobi to design, undertake, and report a trial examining the introduction of clinical guidelines together with training and external supervision to support adoption of these recommended practices in Kenyan district hospitals. Linked to this research work, he created the Emergency Triage, Assessment and Treatment plus Admission Care (ETAT+) course to teach skills in caring for seriously ill children and newborns to doctors, nurses, and paramedics. He and his team helped develop the first, widely distributed evidence-based guidelines (produced as the Ministry of Health ‘Basic Paediatric Protocols’) in 2005. His team coordinated the revision of these guidelines with the Ministry of Health in 2010, in the latter case employing the GRADE approach. Examples of this work and a description of the approach have been published. Formally, Mike English remains a Senior Research Fellow in a research program linking KEMRI and Oxford University with financial support from The Wellcome Trust. In this role he established a formal research collaboration between this program the Ministry of Health and the University of Nairobi aiming to develop capacity in health services research in Kenya. As a representative of the research community, he sits on *ad hoc* working groups when requested by the Kenyan Ministry of Health.

## Supplementary Material

Additional file 1 Outline of how prior, current, and proposed interventions may act as levers to affect uptake of best practice guidelines in Kenyan hospitals based on the levers defined in the Behavior Change Wheel. Click here for file
