# Supplementary material for: Designing a theory-informed, contextually appropriate intervention strategy to improve delivery of paediatric services in Kenyan hospitals
Source: Implement Sci. 2013 Mar 28;8:39. doi: 10.1186/1748-5908-8-39 (PMC3620707; doi:10.1186/1748-5908-8-39)
Supplement: Additional file 1 — Outline of how prior, current, and proposed interventions may act as levers to affect uptake of best practice guidelines in Kenyan hospitals based on the levers defined in the Behavior Change Wheel. [file 1748-5908-8-39-S1.docx]

**Additional File 1:** Outline of how prior, current and proposed interventions may act as levers to affect uptake of best practice guidelines in Kenyan hospitals based on the levers defined in the Behaviour Change Wheel [61].

|  | **Specific Prior Interventions: 2006 - 2010** | **Evolution of intervention and context: 2006 - 2012** | **Ongoing, specific national level interventions: 2013+** | **Specific, targeted intervention package for evaluation: 2013+** |
| --- | --- | --- | --- | --- |
| **Policies*** |  |  |  |  |
| **Communication / Marketing** |  | Discussion of guidelines, uptake and quality of care at multiple professional and policy-linked meetings 2007-2012 | Dissemination of evidence base and guidelines through professional conferences, symposia and other media | Creation of a multi-hospital network as a forum for engaging facilities in adoption of guidelines and improving quality of care |
|  |  | Increasing discussion on quality of care in policy and professional arenas |  |  |
| **Guidelines** | Creation of national paediatric guidelines in 2006 |  | Progressive updating and extension of guidelines in collaboration with policymakers, academics and professional association |  |
|  | Dissemination of 10,000 copies of 2006 guidelines |  | Printing and dissemination of new guideline materials as appropriate and possible |  |
|  | Creation of revised national paediatric guidelines in 2010 employing consensus development conference |  |  |  |
|  | Dissemination of 10,000 copies of 2010 guidelines |  |  |  |
| **Regulation** |  | Adoption of guidelines and promotion by professional association and major medical training institution | Endorsement of existing, revised and new guidelines by professional association and medical training institutions | Developent of consensus based agreements on implementation priorities and goals within network hospitals |
|  |  | Distribution of guidelines during government survey / supervision visits to facilities |  |  |
|  |  | Promotion of clinical audit using guidelines as reference standards |  |  |
| **Service provision** | Initiating discussion on appropriate organisation of care within facilities |  |  | Identification of optimal approaches to service provision that are feasible and appropriate for context within network facilities |
| **Interventions^** |  |  |  |  |
| **Education** | Engaging professionals and policymakers in discussions on evidence and guideline development | Introduction of ETAT+ training into undergraduate and post-graduate paediatric curriculum at University of Nairobi from 2008 | Continued introduction to guidelines and provision of revised ETAT+ training as part of undergraduate and post-graduate paediatric curriculum at University of Nairobi | Improving knowledge and understanding of roles and responsibilities for key professionals leading service provision and enhancing technical knowledge where necessary through a community of practice (CoP) |
| **Training** | Development of ETAT+ training course to improve knowledge and skills in support of guideline use | Progressive but ad hoc increase in ETAT+ training coverage; n > 2500 Trained by June 2012 | Continued efforts to expand access to ETAT+ training in routine hospital settings | Providing key professionals with core skills in management and leadership through a CoP and ongoing learning |
| **Persuasion** |  | Discussion of guidelines, uptake and quality of care at multiple professional and policy-linked meetings 2007-2012 | Continued efforts to enage professionals in discussions on new guidelines and service quality | Development of monitoring and evaluation approaches that provide timely feedback to hospitals and policy makers on implementation of guidelines within network hospitals |
|  |  |  |  | Promotion of reflection on hospital achievements and 'benchmarking' amongst peers linked to shared learning within the CoP |
| **Environmental restructuring** | Initiating discussion on most appropriate use of physical resources / spaces to provide care | Increasing use of 'surveys' and other means to monitor physical and organisatonal capacity to provide services |  | Promotion of reflection on environmental and organisational aspects of service provision and shared learning within the CoP |
| **Modelling** |  | Involvement of over 40 professionals as ETAT+ instructors | Continued engagement of professionals as ETAT+ instructors | Discussion of exemplars within network / CoP and peer to peer visits |
| **Enablement** |  |  |  | Improving skills of negotiation and promoting collective action across network to overcome barriers to improving care |
| **Incentivisation** |  |  |  | Recognition of achievements within network and within professions |
| *** No relevant activities related to the policy areas: Fiscal, Legislation, Environmental / Social Planning** | | | |  |
| **^ No relevant interventions linked to the themes of coercion or restriction** | | |  |  |
